# Impact of Helminth Infections during Pregnancy on Vaccine Immunogenicity in Gabonese Infants

**DOI:** 10.3390/vaccines8030381

**Published:** 2020-07-11

**Authors:** Judith Flügge, Ayôla Akim Adegnika, Yabo Josiane Honkpehedji, Thaisa L. Sandri, Esther Askani, Gédéon Prince Manouana, Marguerite Massinga Loembe, Sina Brückner, Mohamed Duali, Johannes Strunk, Benjamin Mordmüller, Selidji Todagbe Agnandji, Bertrand Lell, Peter G. Kremsner, Meral Esen

**Affiliations:** 1 Institute for Tropical Medicine, University of Tübingen, 72074 Tübingen, Germany; judith.fluegge@uni-tuebingen.de (J.F.); aadegnika@cermel.org (A.A.A.); thaisa.lucas-sandri@uni-tuebingen.de (T.L.S.); esther.askani@hotmail.com (E.A.); manouanacermel@gmail.com (G.P.M.); loembeM@africa-union.org (M.M.L.); sinab@gmx.net (S.B.); johannes.strunk@web.de (J.S.); benjamin.mordmueller@uni-tuebingen.de (B.M.); peter.kremsner@uni-tuebingen.de (P.G.K.); 2 German Center for Infection Research (DZIF), Partner Sites, 72074 Tübingen, Germany; bertrand.lell@cermel.org; 3 Centre de Recherches Médicales de Lambaréné (CERMEL), B.P. 242 Lambaréné, Gabon; hyjosy@gmail.com (Y.J.H.); dualimoh@yahoo.fr (M.D.); agnandjis@cermel.org (S.T.A.); 4 Department of Parasitology, Leiden University Medical Center, 2333 ZA Leiden, The Netherlands; 5 Department of Medicine I, Division of Infectious Diseases and Tropical Medicine, Medical University of Vienna, 1090 Vienna, Austria

**Keywords:** parasite infection, helminths, vaccine immunogenicity

## Abstract

Helminth infections are common in sub-Saharan Africa. Besides direct clinical effects, a bias towards a T helper type 2 (Th2) cell immune response is observed. The consequences of parasite infection during pregnancy for the mother and particularly for the fetus and the newborn can be severe and may include impaired immune response during acute infection and vaccination. Here, we present data of immune responses to vaccines given within the expanded program on immunization (EPI) of infants born to helminth infected or non-infected mothers. The study was conducted in Lambaréné and surroundings, Gabon. Maternal helminth infection was diagnosed microscopically using the Kato-Katz method for soil-transmitted helminths (STH), urine filtration for *Schistosoma haematobium* infections and the saponin-based method for filarial infections. Plasma antibody levels to different vaccine antigens were measured in mothers and their offspring by enzyme-linked immunosorbent assay (ELISA) at different timepoints. We found 42.3% of the mothers to be infected with at least one helminth species. Significantly lower anti-tetanus toxoid immunoglobulin (Ig) G was detected in the cord blood of infants born to helminth infected mothers. Following vaccination, immune responses of the infants to EPI vaccines were similar between the two groups at nine and 12 months. Even though infection with helminths is still common in pregnant women in Gabon, in our setting, there was no evidence seen for a substantial effect on infants’ immune responses to vaccines given as part of the EPI.

## 1. Introduction

Through the implementation of vaccination programs like the expanded program of immunization (EPI), infants worldwide receive very similar vaccination schemes. However, there are tremendous discrepancies within and between different regions regarding vaccine efficacy with several studies reporting that children in low- and middle-income countries are less responsive to vaccines than children from high-income countries [1,2,3,4,5,6,7,8]. This is presumably associated with malnutrition and infections, in particular with parasites [9,10,11,12].

Helminth infection affects more than two billion people worldwide [13,14] and is highly prevalent in sub-Saharan Africa. It is considered as a major public health problem but remains neglected because helminths mostly affect underprivileged populations of low- and middle-income countries. However, children resident in several African regions are at high risk to acquire helminth infection, which can cause various symptoms that may lead to significant morbidity like weight loss, growth impairment [15], anemia [16,17], and diarrhea, ultimately ending up in physical and cognate retardation, reduced school performance and impaired daily life [18,19,20]. In different areas of Gabon, recent studies suggest a high pervasiveness of helminth infections. Two publications present an infection prevalence of more than 50% in their study population. Nguema et al. examined the prevalence of soil-transmitted helminths (STH) and schistosomiasis among schoolchildren of the Northern and Eastern Health regions of Gabon [21] and Veletzky et al. found a prevalence of 50.8% of the filarial parasite *Loa loa* among the 1232 participants from the Gabonese departments of Tsamba-Magotsi and Ogooué et des Lacs [22]. Studies conducted in our study area have assessed incidences of STH and schistosomiasis ranging from 65% in a study with 388 pregnant women in 2010 [23] to 23% of schistosomiasis infections in a smaller study with 54 pregnant women in 2019 [24] and 26% for schistosomiasis as well as 15% for STH in 614 schoolchildren living in Lambaréné in 2020 [25].

Helminths are thought to modulate the host immune system towards T helper type 2 (Th2) cell responses by reducing the inflammatory immune response and inducing a more tolerant phenotype through regulatory T-lymphocytes and anti-inflammatory cytokines [26,27]. This altered immune pattern could be protective by reducing the risk of immune-mediated diseases [28]. In this context, it has been shown that helminth-infected individuals rarely have allergic reactions to parasites and suffer less from allergic disorders and inflammatory diseases compared to helminth-free individuals [29,30,31,32,33]. On the other hand, it has been shown that the immune response towards viral infections in humans can be reduced [34]. In addition, in a model system helminth infection suppressed immune responses to an influenza vaccine by regulatory mechanisms [35]. Hence, in both, in the mouse model and in humans, helminth infection may have strong bystander effects [8].

Previously, we have observed that children infected with *Trichuris trichiura* had lower antibody responses against the malaria vaccine candidate GMZ2 (a fusion protein of *Plasmodium falciparum* glutamate-rich protein (GLURP) and merozoite surface protein 3 (MSP3) than uninfected children or children infected with *Ascaris lumbricoides* [36]. Additionally, other studies have shown that infection with intestinal parasites could influence the immunological outcome of vaccination [4,37]. Cooper et al. have reported that anthelminthic treatment prior to an oral cholera vaccination results in enhanced immune responses to the vaccine [38], which could advocate an interventional strategy to improve vaccine responses. However, in the setting of one of our previous studies performed with school children aged 6–10 years, a single dose of an anthelminthic treatment prior to vaccination had only subtle effects on vaccine immunogenicity [39,40].

It is hypothesized that newborns of helminth infected mothers are exposed to parasite-derived products that seem to cross the placenta and potentially prime or tolerate the fetal immune response to parasite-specific as well as unrelated antigens [41,42]. This early exposure may have long-term effects on the child’s immune system, including their response to immunization [43,44]. However, several studies have not provided evidence that vaccine-induced immune responses of infants are strongly affected by the helminth infection status of the mother [45,46,47,48].

The aim of the present study was to systematically assess vaccine immunogenicity to EPI vaccines in newborns born to helminth-infected and helminth-free mothers in Lambaréné, Gabon.

## 2. Materials and Methods

### 2.1. Study Design and Participants

Healthy, pregnant women and their newborns from Lambaréné and surroundings in Gabon were enrolled to be part of this study, which was conducted between February 2014 and February 2017. Four visits were scheduled for all subjects. Visit (V) 1 took place within the last four weeks of pregnancy (mothers only), V2 at delivery (mothers and their newborns), V3 nine months after delivery (infants only) and V4 12 months after delivery (infants only). At V1, stool, urine, and blood samples of the mother were collected to assess helminth infections. At delivery, blood samples of the mother and her newborn (cord blood) were collected to assess antibody baseline levels of the infants. At V3 and V4, blood of the infant was collected to assess antibody levels to the given EPI vaccines (tetanus, diphtheria, pertussis, *Haemophilus influenzae* type b (HiB), poliomyelitis (polio), hepatitis B, and measles). Written informed consent was obtained from all participating mothers.

Inclusion criteria were good general health upon clinical examination, willingness to deliver in one of the two maternities in the study area, willingness to give blood for the examinations and to provide samples for helminth assessment (stool, urine, and blood for filarial diagnostics). Furthermore, the participants should have been resident in the area until the end of the study.

Exclusion criteria were the participation in another clinical trial interfering with the present study and any confirmed or suspected immunosuppressive or immunodeficient condition resulting from disease (e.g., human immunodeficiency virus (HIV) infection). Women with known chronic disease and chronic infection (hepatitis B or tuberculosis) were also excluded. Women with signs of symptoms of helminth infection were not included, meaning only participants with asymptomatic helminth infection were included. A further exclusion criterium was being not resident in the area until the end of the study.

### 2.2. Vaccination Schedule

During the study, infants received the vaccines of the EPI: Bacillus Calmette-Guérin (BCG) vaccine (Serum Institute of India Pvt. Ltd., Pune, India) and polio (Biopolio^®^ B1/3, Bharat Biotech International Ltd., Hyderabad, India) at birth; DTwP-rHepB–Hib (Liquid) Pentavalent compromising Diphtheria, Tetanus, Pertussis (Whole cell), Hepatitis B (rDNA) and HiB conjugate Vaccine (Adsorbed) (Biological E. Limited, Hyderabad, India) at 6, 10, and 14 weeks of age and additional polio at 14 weeks of age. Measles vaccine (Serum Institute of India Pvt. Ltd., Pune, India) and Yellow fever vaccine (FSBSI “Chumakov FSC R&D IBP RAS”, Moscow, Russia) were administered at nine months of age. The vaccination took place in the vaccination center at the antenatal care center of the study areas.

### 2.3. Assessment of Parasite Infection

Stool, urine, and blood samples were collected from the mothers at V1 to assess helminth infection. One stool sample was analyzed for the presence of soil-transmitted helminths using standard methods including Kato-Katz method [49,50], three consecutive urine samples were analyzed using the filtration method to detect the ova of *S*. *haematobium* [51] and blood samples were analyzed to detect filaria larvae using the saponin method [52]. All methods are described in more detail in the following.

Two Kato-Katz preparations were made for each homogenized stool sample and microscopically read by two independent readers within 120 min following arrival of the sample at the laboratory and including the preparation and the second reading of each slide, especially for the time-limited detection of hookworm eggs. Two more readings were done after 24 hours. The results were recorded as the number of eggs per slide. In case of any discrepancy, a third reading, blinded to the results of the first and second reading, was performed. The methods to detect the ova of *S. haematobium* consisted in its filtration of 10 mL or a specific volume of urine sample through the Millipore filter that concentrate eggs on the filter membrane. Two independent readers microscopically examined the entire filter.

The utilized saponin-based technique was performed to detect the microfilaria larvae in 1 mL or a precise volume of blood that was dispensed into 2% saponin lysing solution. After 10 min centrifugation at 2000 rpm, the entire preparation was examined by two independent readers.

### 2.4. Assessment of Vaccine-Specific Antibody Levels (Immunoglobulin IgG)

Plasma antibody levels to the EPI vaccines were measured using commercial and validated quantitative enzyme-linked immunosorbent assay (ELISA) kits. Analysis was performed with samples from mothers and infants whose helminth status was known and who have completed V1 to V3 and V4 for the analysis of antibodies against the measles vaccine.

The utilized ELISA kits were as follows: Human anti-hepatitis B virus surface antibody (HBsAb) ELISA Kit (CUSABIO Biotech Co., Houston, TX, USA), Haemophilus influenzae B IgG ELISA (Hölzel Diagnostika GmbH, Cologne, Germany), Corynebacterium diphtheriae toxin IgG ELISA (GenWay Biotech, Inc., San Diego, CA, USA), Poliomyelitis Virus IgG ELISA (GenWay Biotech, Inc., San Diego, CA, USA), Tetanus Toxoid IgG ELISA (GenWay Biotech, Inc., San Diego, CA, US), Bordetella pertussis IgG ELISA (GenWay Biotech, Inc., San Diego, CA, USA), and Anti-Measles virus IgG Human ELISA Kit (Abcam, Cambridge, UK). Specifically, for anti-hepatitis B levels, the calculation of IgG is shown here as fold-difference based on the ratio of the optical density (OD) measured in the sample and the OD of the negative sample provided by the kit (OD sample/OD negative) as indicated by the manufacturer.

The assays were conducted according to the manufacturer´s recommendations. Absorbance was measured at 450 nm using a Photometer (Phomo, Anthos Mikrosysteme GmbH, Krefeld, Germany). Antibody concentration was determined using the reference curve generated with the standards provided in each kit.

### 2.5. Ethical Approval

All subjects gave their informed consent for inclusion before they participated in the study. The study was conducted in accordance with the Declaration of Helsinki and approved by the “Ethik-Kommission an der medizinischen Fakultät der Eberhard-Karls-Universität und am Universitätsklinikum Tübingen” (253/2013BO1) as well as by the “Comité d´Ethique institutionnel du CERMEL” (CEI-CERMEL Nº13/2013).

The study is registered at ClinicalTrials.gov (Identifier: NCT02714348).

### 2.6. Statistical Analysis

Normal distribution of antibody titers was assessed by D’Agostino & Pearson and Shapiro–Wilk normality tests. Chi-square test or Fisher’s exact test were used for categorical variables, whereas the Mann–Whitney test was used to compare medians. Spearman’s rank test was used to investigate the relationship between immunological markers and clinical continuous variables. Categorical data were presented as counts and percentages, and continuous data were expressed as median and interquartile range. For all the analysis, groups were formed as mothers, cord blood, infants with 9 and 12 months old and the antibody levels compared according to infant’s gender (female versus (vs.) male and mothers/cord blood of females vs. mothers/cord blood of males) and helminth infection state (no helminth infection and helminth infection). Helminth infection was subsequently subcategorized and compared regarding the multiplicity of infection (no helminth infection, single helminth infection and multiple helminth infection), regarding the infective helminth species (*Trichuris* sp. infection, *Schistosoma* sp. infection, and other helminths). Outliers were removed following the ROUT method. The significance level considered was *p* < 0.05 and all the analyses were performed using GraphPad Prism (version 7.0a, GraphPad Software, San Diego, CA, USA).

## 3. Results

A total of 323 mothers were enrolled in the trial. Samples of all four visits (V1, V2, V3, and V4) were available for 58 of the participants. For another 65 mother-child pairs three samples were available, so in total 123 mothers and their infants were evaluated during the final analysis.

### 3.1. Baseline Characteristics

The assessment of parasite infection showed that 42.3% (52/123) of the enrolled mothers were helminth positive. The prevalence of *S. haematobium* was the highest, occurring in 26.9% (14 in 52) of the infected mothers. Additionally, 7.7% (4/52) of the mothers were positive for *L. loa*. Regarding the intestinal helminths, *T. trichiura* was found in 13.5% (7/52), hookworm in 7.7% (4 in 52), and *A. lumbricoides* in 5.8% (3/52). *Fasciola hepatica*, *Strongyloides stercoralis*, and *Enterobius vermicularis* were found infecting one woman (1.9%) each. A total of 28.8% (15/52) of the infected women had co-infections with two or more of the named parasites and no infection was found in 57.7% (71 in 123) of all investigated mothers.

The median age of women at enrollment was 24 years. For 22.8% of the women, this was their first gestation, while 35% reported having had 2–4 previous parities and 17% reported five or more. The median birth weight of children was 3.1 kg (Table 1).

### 3.2. Assessment of Vaccine-Specific IgG Levels

#### 3.2.1. Polio

**Figure 1:**
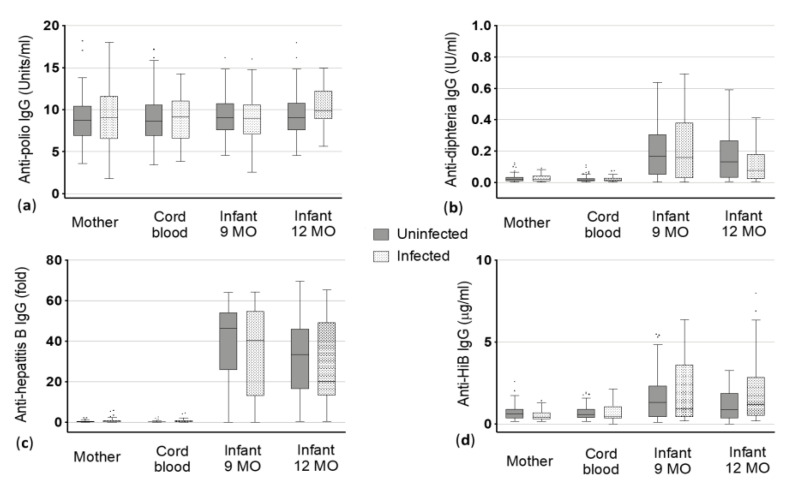
Vaccine-specific immunoglobulin (Ig) G levels to (**a**) poliomyelitis (polio), (**b**) diphtheria, (**c**) hepatitis B and (**d**) *Haemophilus influenzae* type B (HiB) antigens in maternal blood and cord blood at delivery and the infants at 9 and 12 months (MO) of age. Dots represent the outliers not removed by ROUT method.

The median anti-polio IgG levels were 8.74 in helminth negative women and 9.01 Units/mL in helminth infected women. As expected, corresponding cord blood IgG levels did not significantly differ when compared with the mothers (Figure 1, Table 2). Additionally, no significant difference in pre and post-vaccination titers of infants born to helminth-infected and non-infected mothers was observed. At nine months of age, levels of infants born to uninfected mothers and those born to infected mothers were almost similar (9.01 and 9.0 Units/mL, respectively, Table 2). Similar levels were also observed for the infants at 12 months of age with 9.04 Units/mL for those born to uninfected mothers and 9.83 Units/mL for infants born to infected mothers.

#### 3.2.2. Diphtheria

The median antibody levels against diphtheria toxoid were similar in uninfected mothers and infected mothers as well as in the corresponding cord blood (median 0.02 International Units (IU)/mL for all, Figure 1, Table 2). Irrespective of helminth infection, the anti-diphtheria IgG levels increased in children at nine months of age (median 0.17 IU/mL in children of uninfected mothers vs. 0.16 IU/mL of infected mothers). At one year of age, the IgG levels decreased in general, but children of uninfected mothers had slightly but not significantly higher median levels compared to children born to infected mothers (0.13 IU/mL vs. 0.08 IU/mL) (Figure 1, Table 2).

#### 3.2.3. Hepatitis B

Mother and the corresponding cord blood showed, as expected, almost similar IgG levels against hepatitis B antigens in the uninfected (0.24 and 0.18 fold) and infected group (0.32 and 0.24 fold). As the cut-off for positivity in this ELISA was > 2.1, mothers and cord blood were considered as not being positive for anti-hepatitis B antibodies (Figure 1). In contrast to that, highly positive levels were found when looking at infants after vaccination. Additionally, a clear difference was seen between the children of uninfected mothers and children born to infected mothers at nine and 12 months of age in the median anti-hepatitis B IgG levels. At nine months, children of uninfected mothers showed a median level of 46.41 fold vs. 40.45 fold in children of infected mothers. More distinct was the difference between children of uninfected mothers and those of infected mothers at 12 months: children born to uninfected mothers had a median IgG level of 33.43 fold whereas children born to helminth infected mothers could only reach a median of 20.15 fold (Figure 1, Table 2). However, these differences were not statistically significant.

#### 3.2.4. HiB

Median IgG levels against HiB vaccine antigens were 0.37 and 0.39 µg/mL in mother and cord blood of the uninfected group, respectively. With median basic antibody titers against HiB vaccine antigens of 0.31 and 0.36 µg/mL maternal blood and cord blood of the helminth infected group, respectively, showed no significant difference compared to the uninfected group (Figure 1, Table 2). Children born to helminth uninfected mothers (median: 0.43 µg/mL) had similar IgG levels like children born to infected mothers (median: 0.44 µg/mL) at the age of nine months. In contrast, children at 12 months of age had clearly, but not significantly lower antibody titers when born to uninfected mothers compared to children born to helminth infected mothers (medians 0.35 vs. 0.51 µg/mL).

#### 3.2.5. Pertussis

**Figure 2:**
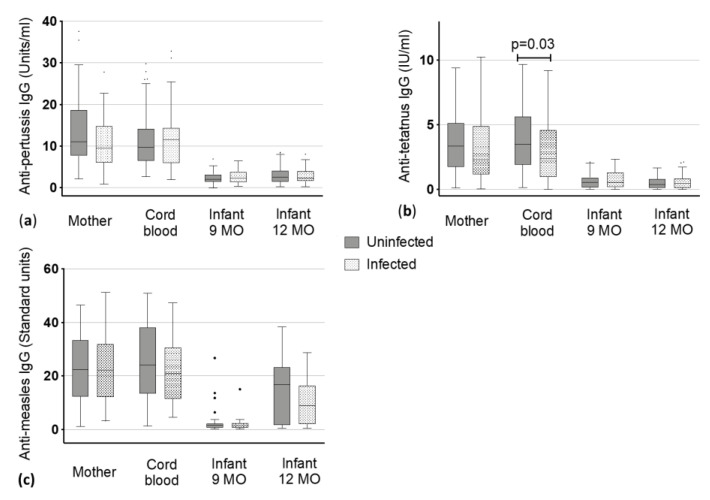
Vaccine-specific immunoglobulin (Ig) G levels to (**a**) pertussis, (**b**) tetanus and (**c**) measles antigens in maternal blood and cord blood at delivery and the infants at 9 and 12 months (MO) of age. Dots represent the outliers not removed by ROUT method.

Anti-pertussis median IgG levels of mother and cord blood ranged from 9.40 to 11.54 Units/ml without significant difference between being uninfected or infected (median mothers: 11 vs. 9.4 Units/ml and median cord blood: 9.64 vs. 11.54 Units/mL, Figure 2). IgG in children declined within V3 and V4 to around 2 Units/mL with similar levels in children at nine (1.99 vs. 2.35 Units/mL) and 12 months of age (2.5 vs. 2.31 Units/mL) and there was no significant difference between children born to uninfected mothers and children born to infected mothers was detected (Figure 2).

#### 3.2.6. Tetanus

Levels of uninfected mothers and the corresponding cord blood had slightly higher values than infected mothers and corresponding cord blood. Uninfected mothers had a titer of 3.37 Units/mL whereas infected mothers showed a titer of 2.31 (Figure 2, Table 2). A statistically significant lower level of antibodies was found in the cord blood of infants born to infected mothers when compared to those of uninfected mothers (2.38 vs. 3.48 Units/mL, *p* < 0.03) (Figure 2). The IgG levels against tetanus toxoids declined from infants being nine months old to three months later at 12 months of age (from 0.54 (nine months) to 0.39 Units/mL (12 months) in children of uninfected mothers and from 0.54 (nine months) to 0.47 Units/mL (12 months) in children of infected mothers). However, these changes were not significantly different between children born to uninfected mothers or infected mothers.

#### 3.2.7. Measles

Antibodies against measles virus antigens were present in both groups, the non-infected mothers (median: 22.39 Standard units) and the helminth infected mothers (median: 22.14 Standard units) with no significant difference in the IgG level between the two groups. As expected, we did not detect a difference in the corresponding cord blood. Accordingly, there was no difference in baseline IgG against measles-antigens between the babies of the uninfected and infected mothers. At nine months, almost no antibodies against measles could have been detected in infants (1.6 vs. 1.46 Standard units), but these increased following the measles vaccine that is given at month 9 (median in 12 months old: 16.75 vs. 8.89 Standard units). No significant difference was detected in infants born to uninfected or infected mothers at the two timepoints (Figure 2, Table 2).

### 3.3. Additional Considerations

Median IgG levels and interquartile values (25th and 75th percentiles) to the EPI vaccines of mother, cord blood and children at 9 and 12 months of age (mothers uninfected vs. infected vs. any single helminth infection vs. multiple helminth infections vs. a single *Trichuris* sp. infection vs. a single *Schistosoma* sp. infection) are presented in Table 2.

**Figure 3:**
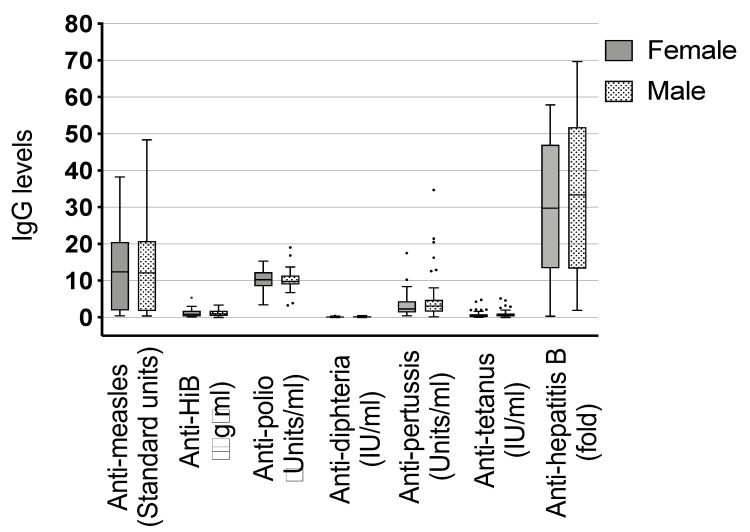
Comparison of immunoglobulin (Ig) G to expanded program of immunization (EPI) vaccines between female and male infants. Dots represent the outliers not removed by ROUT method.

Other antibody titer correlations tested were the following: female vs. male children (Figure 3), children who have been breastfed vs. those who have not been breastfed, mothers with a single helminth infection vs. multiple infections and the two most common helminths in our setting against each other and vs. single and multiple infections: mothers infected with *Schistosoma* sp. vs. mothers infected with *Trichuris* sp., *Schistosoma* sp. infected mothers vs. any single helminth infection, and *Schistosoma* sp. infected mothers vs. mothers with multiple infections and the same correlations for *Trichuris* sp. infected mothers (data not shown). We did not detect a difference between any of these comparisons.

## 4. Discussion

The current study was designed to assess a potential influence of maternal helminth infection during pregnancy on the infant’s immune response to standard vaccines given within the EPI. It has been shown that helminth infection in general influences the immune system of the host [4,36,37,38,53] and, more specifically, that maternal helminth infections prime the fetal immune system to parasite specific antigens and unrelated antigens. Theoretically, this could have an effect on vaccination efficacy [41,42,43,44]. Nevertheless, in our study setting, we found no evidence that maternal helminth infection during pregnancy is influencing the immune response of the infant to EPI vaccine antigens. Besides a trend towards lower antibody titers of children whose mothers were infected with helminths, both groups had very similar responses.

These findings are consistent with other recent publications that found no or only minor effects of helminth infection on immune responses to vaccines [54,55]. Webb et al. [46] as well as Nash et al. [45] also tested children at one year of age for antibody levels against tetanus and measles immunization and found no effect of a maternal anthelminthic treatment before birth on child’s immunogenicity. A publication from Ondigo et al. [48] shows that maternal infection with schistosomes during pregnancy had no effect on the infant’s antibody levels to tetanus and diphtheria toxoid. Interestingly, they observed that later on, at the age of two years, children developed a significantly lower mean antibody level to their recent measles immunization (given regularly at month 9) when born to an infected mother. However, at two years of age, it is possible that children already have acquired a helminth infection which could have biased the outcome of these immune measurements. We have not screened the children for a helminth infection. Hence, undiagnosed helminth infection in infants or children could have potentially affected our results. However, as it is not likely that helminth infections occur before nine months of age [53], which represents the timepoint of our first IgG measurement in the children (following baseline investigation in cord blood), we assume that helminth infections are not a strong bias for our results.

In our study the children born to helminth infected mothers also had lower measles specific antibody titers at 12 months of age, three months following the measles vaccine, but the difference was not significant (Figure. 2, Table 2). This difference could potentially be significant later on and therefore screening young children for helminths at a later timepoint should be considered in future studies.

Another study evaluated the immune responses of adult individuals with and without schistosome infection at the timepoint of hepatitis B vaccination and tetanus toxoid boosts. They also found no impact of this helminth infection on the production of corresponding antibodies towards vaccine antigens [56].

Our only significantly lower values were detected for anti-tetanus toxoid IgG in the cord blood of infants born to helminth infected mothers (Figure 2, Table 2). Interestingly, this was not observed for other vaccine antigens. It has to be taken into account that during pregnancy tetanus vaccination is highly recommended to protect the newborn from neonatal tetanus infection and therefore, most pregnant women in Gabon receive a tetanus vaccination. We did not evaluate how many mothers had a tetanus booster vaccination during their pregnancy in our study setting. However, due to the highly elevated antibody titers of the mothers, we assume that most of the participants got the booster. This topic will be discussed further in more detail.

In the present study, the infant’s antibody levels measured following vaccination only show a trend towards reduced immune responses, i.e., for measles, hepatitis B and diphtheria. The lack of significance could be due to the relatively small sample size of 123 mother-child pairs further categorized in the two groups “infected” and “uninfected”, although it shall provide enough power to detect a clinically significant effect. Additionally, other limitations could have impacted the outcome of our investigations. Thus, there could be errors in analyzing helminths by microscopy, leading to false positive or negative results which could be attributed to (i) not analyzing three consecutive stool samples, (ii) the quality of the samples, e.g., if it was not possible to prepare and read all samples within a very early time frame, or (iii) misinterpretation by the readers. Furthermore, we might have lacked the detection of some other intestinal pathogens by not using more specific and sensitive techniques like formol-ether concentration, stool culture and ideally PCR.

Alternatively, women could have taken an anthelminthic treatment before the investigation leading to negative microscopic detection, but the immune system has not changed until the time point of investigation. In our setting it was difficult to investigate whether anthelminthic treatment was taken during pregnancy. It is recommended that pregnant women receive a deworming treatment in early pregnancy, but women do not always take the medication for different reasons like underestimating the risk of helminth infection during pregnancy and low acceptance of medication intake during pregnancy. This is an important topic for future studies employing guided and standardized interviews. Considering that a single-dose anthelminthic treatment is sufficient to reduce the intensity of (or clear) some but not all helminth infections [57] and that we might have missed some infections due to methodological limitations, we assume that the number of women who took anthelminthic treatment is equally distributed in helminth positive as well as in helminth negative tested volunteers. Nonetheless, it is possible that the number of infected individuals could have been underestimated due to these limitations.

Concerning the infant’s vaccine-specific antibody levels, there was no increase in infant’s tetanus and pertussis IgG levels following EPI vaccinations in contrast to anti-measles, hepatitis B and diphtheria related IgG. Instead, a decrease was observed when comparing the data with the baseline titer represented by cord blood measurements. The median anti-pertussis IgG levels of mother and cord blood were above 10 Units/mL and showed a remarkable decrease at the age of nine months to around 2 Units/mL which remained stable until one year of age. For median anti-tetanus toxoid IgG levels, it was similar. Maternal and cord blood had above 2 IU/mL and the levels of the corresponding children declined after vaccinations to about 0.5 IU/mL, as shown in Figure 2. This could be due to several reasons. The vaccination may not have been efficacious, but in our assay the level of protection against tetanus is indicated at 0.01 IU/mL [56], so the children’s levels are above the cut-off for protection, which means the vaccination should have been effective. In this context, it is important to note that we did not check for the quality of antibodies, since we have not performed avidity tests and killing assays for bacteria and viruses, nor did we check for the different Ig isotypes. We analyzed exclusively the quantity of IgG antibodies following vaccination, but antibody quality should be also considered in future studies.

To explain the high anti-tetanus and anti-pertussis levels of maternal and corresponding cord blood in case of tetanus is simple, because according to the local antenatal healthcare centers recommendation, mothers receive a booster vaccination of tetanus toxoids during pregnancy to prevent neonatal tetanus as mentioned above. We could not truly analyze how many mothers received a booster vaccine because the women usually did not carry a vaccination card with them and sometimes, they did not know whether they have received the recommended tetanus vaccination or not. According to the collected data, only 14 women from our cohort had an IgG titer below 1 IU/mL (data not shown), suggesting that they did not receive a tetanus booster but still are protected, because their titers were above the protective titer of 0.1 IU/mL. Our results encourage us to investigate the acceptance and realization of recommended vaccines in more detail in future studies.

At the moment, vaccination against pertussis during pregnancy is not recommended in Gabon, but an explanation for the high maternal anti-pertussis IgG levels could be that the mothers came in contact and got infected during their lifetime with *Bordatella* spp. which are still responsible for thousands of deaths in sub-Saharan Africa every year especially in infants [58]. The low anti-pertussis IgG concentrations of the children raise the question of whether transferred maternal antibodies could inhibit the generation of autologous antibodies in infants [59]. Studies from North America, Belgium, and England detected lower antibody responses following vaccination in infants whose mothers received vaccines during pregnancy [60,61,62]. However, the significance of this effect remains unknown and, in several countries like Australia, United Kingdom, United States, Belgium, Spain, and just recently, Germany, maternal pertussis vaccination during pregnancy is recommended. Studies could show that the vaccine efficacy in infants, born to vaccinated mothers, who are below three months of age was up to 90.9% and reduced the pertussis-related infant deaths significantly [63,64,65]. Nonetheless, the survey from England reported that the protection from maternal immunization lasts until the first infant vaccination dose but not until the third dose. It should be further investigated and evaluated whether it makes sense to start immunization of infants in general at a timepoint when it is likely that maternal antibodies are present in high amounts and/or to consider a boost of tetanus and pertussis vaccine together with the measles and yellow fever vaccine at nine months of age, especially in highly endemic areas.

We also checked for differences in vaccine-specific IgG between male and female children because gender aspects become increasingly important. Females are known to respond with higher antibody production to some vaccinations than males [66,67]. Sex-specific immune response was shown for several vaccines like influenza, yellow fever, measles, mumps, and rubella. The authors report that these results were also seen when vaccinating at different timepoints, in childhood or adulthood [68]. Possible reasons could be of genetic, immunological or hormonal origin which differs between sex. Even behavioral differences may also contribute to the outcome of vaccination. Nevertheless, the authors also state that most studies do not report sex-specific effects on vaccine immunogenicity, which could be due to the sex being not considered as a significant variable or that in these studies the investigators have not found differences. In our study, we could not detect any differences in vaccine-specific IgG when comparing male and female children. Furthermore, we explored other correlations, e.g., concerning the effect of single and multiple infections and the species of helminth infection. None showed a significant association.

## 5. Conclusions

In our study setting, infection with helminths in pregnant women in Gabon is still common, but does not affect the infant’s immunogenicity to vaccines of the routine EPI. This result could be due to some study limitations, like small sample size, methodology of parasite assessment, or children not being screened for helminth infection. On the other hand, it seems that the infant’s immune response towards vaccines is not significantly affected by maternal helminth infection, like it was shown by several other studies, and if affected, this may be balanced by other factors that were not investigated in this study.

## Figures and Tables

**Table 1:** Baseline characteristics of mothers and their infants overall and considering the helminth infection status of the mothers.

Characteristic	Overall	Uninfected	Infected
**Mother (n)**	123	71	52
Median age in years (range)	24 (13–43)	26 (13–42)	23 (14–43)
Median weight in kg (range)	63 (37–153)	65 (45–153)	63 (37–100)
Median height in cm (range)	160 (141–171)	160 (147–171)	159 (141–170)
Parity			
1st parity in % (n)	22.8 (28)	18.3 (13)	28.8 (15)
1 previous parity in % (n)	18.7 (23)	18.3 (13)	19.2 (10)
2-4 previous parities in % (n)	35 (43)	38 (27)	30.8 (16)
5 or more previous parities in % (n)	17 (21)	21.1 (15)	11.6 (6)
NA in % (n)	6.5 (8)	4.3 (3)	9.6 (5)
**Infant (n)**	124	71	53
Median birth weight in kg (range)	3.1 (1.7–4.3)	3.1 (1.7–4.1)	3.1 (2.1–4.3)
Median height in cm (range)	50 (43–57)	50 (44–56)	50 (43–57)
Sex *			
Male (n)	61	33	28
Female (n)	59	35	24
Breastfeeding			
Yes (n)	48	25	23
No (n)	52	32	20
NA (n)	24	14	10

*: sex was not reported for 04 infants.

**Table 2:** Median values and interquartile values (25th and 75th percentiles) of vaccine-specific immunoglobulin (Ig) G levels in helminth uninfected and infected mothers, corresponding cord blood and their infants at nine months (MO) and 12 months of age. Anti-polio IgG in Units/mL; anti-diphtheria IgG in IU/mL; anti-hepatitis B IgG in fold; anti-*Haemophilus influenzae* type B (HiB) IgG in µg/mL; anti-pertussis IgG in Units/mL; anti-tetanus IgG in IU/mL; anti-measles IgG in Standard Units. Infection with helminths is related to mothers only, as infants were not screened for infection.

	**Mother**						**Cord Blood**					
	**uninfected**	**infected**	**single infection**	**multiple infection**	***Trichuris* sp.**	***Schistosoma* sp.**	**uninfected**	**infected**	**single infection**	**multiple infection**	***Trichuris* sp.**	***Schistosoma* sp.**
**Polio**	8.74 (6.86–10.48)	9.01 (6.53–11.61)	9.01 (6.50–11.67)	8.35 (6.53–10.03)	9.69 (9.69–12.11)	8.69 (6.02–10.08)	8.61 (6.88–10.59)	9.11 (6.57–11.06)	9.09 (5.56–10.93)	9.41 (6.31–11.91)	9 (6.06–9.89)	8.40 (6.12–10.34)
**Diphtheria**	0.02 (0.01–0.03)	0.02 (0.01–0.04)	0.02 (0.01–0.05)	0.0 6(0.01–0.11)	0.03 (0.01–0.06)	0.02 (0.01–0.06)	0.02 (0.01–0.03)	0.02 (0.01–0.03)	0.02 (0.004–0.04)	0.03 (0.01–0.14)	0.02 (0.01–0.05)	0.01 (0.006–0.04)
**Hepatitis B**	0.24 (0.09–0.57)	0.32 (0.15–1.07	1.07 (0.19–24.40)	0. 66(0.15–6.173)	0.31 (0.15–24.40)	0.47 (0.15–2.37)	0.18 (0.11–0.44)	0.24 (0.10–0.44)	0.50 (0.22–26.05)	0.27 (0.11–6.13)	0.27 (0.20–12.75)	0.33 (0.13–2.09)
**HiB**	0.37 (0.16–0.63)	0.31 (0.16–0.43)	0.45 (0.34–0.79)	0.83 (0.40–1.24)	0.55 (0.35–1.25)	0.79 (0.38–1.90)	0.39 (0.17–0.59)	0.3 6(0–0.49)	0.48 (0.34–0.90)	0.78 (0.38–1.16)	0.92 (0.45–1.61)	0.84 (0.40–1.24)
**Pertussis**	11 (7.68–18.68)	9.40 (6.08–14.74)	8.82 (4.24–14.68)	11.82 (8.81–15.95)	11.82 (5.77–14.67)	10.1 6(5.32–14.93)	9.64 (6.43–14.16)	11.54 (5.97–14.33)	11.40 (5.40–12.58)	13.0 6(8.13–23.28)	11.53 (7.89–13.10)	11.69 (6.24–18.97)
**Tetanus**	3.37 (1.76–5.14)	2.31 (1.17–4.91)	2.72 (1.42–4.84)	1.75 (0.85–5.09)	2.74 (1.52–5.29)	1.68 (0.75–3.36)	3.48 (1.90–5.64)	2.38 (0.96–4.59)	2.74 (1.18–4.90)	1.91 (0.56–4.15)	3.05 (1.59–5.32)	1.79 (0.98–3.63)
**Measles**	22.39 (12.21–33.45)	22.14 (12.14–31.93)	22.14 (12.72–31.95)	14.04 (9.5–30.13)	25.15 (13.26–30.13)	17.22 (10.58–30.59)	24.20 (13.45–38.12)	20.94 (11.50–30.57)	23.99 (14.6–33.05)	16.22 (9.98–25.96)	23.78 (14.52–34.01)	16.65 (10.55–34.33)
	**Child 9 MO**						**Child 12 MO**					
	**uninfected**	**infected**	**single infection**	**multiple infection**	***Trichuris* sp.**	***Schistosoma* sp.**	**uninfected**	**infected**	**single infection**	**multiple infection**	***Trichuris* sp.**	***Schistosoma* sp.**
**Polio**	9.01 (7.53–10.76)	9 (7.07–10.63)	8.83 (6.58–10.32)	9.19 (7.48–10.80)	9.38 (7.42–10.80)	9.2 (5.40–11.34)	9.04 (7.54–10.82)	9.83 (8.87–12.25)	9.69 (8.22–11.58)	9.97 (8.82–12.25)	9.83 (8.46–12.36)	9.69 (8.09–12.33)
**Diphtheria**	0.17 (0.05–0.31)	0.1 6(0.03–0.27)	0.17 (0.03–0.37)	0.05 (0.02–0.40)	0.05 (0.01–0.42)	0.14 (0.03–0.36)	0.13 (0.31–0.27)	0.08 (0.02–0.18)	0.11 (0.02–0.19)	0.08 (0.02–0.49)	0.1 6(0.03–0.38)	0.09 (0.04–0.34)
**Hepatitis B**	46.41 (25.75–54.19)	40.54 (13.15–54.86)	43.95 (21.48–55.35)	28. 66(9.85–55.33)	22.82 (5.02–54.96)	38.91 (12–53.17)	33.43 (16.43–46.21)	20.15 (13.18–49.28)	35.31 (7.35–48.77)	17.28 (10.78–49.65)	16.14 (5.99–49.65)	19.32 (5.95–45.27)
**HiB**	0.43 (0.11–1.31)	0.44 (0.19–0.93)	0.91 (0.42–2.64)	1.50 (0.41–4.93)	0.90 (0.29–4.02)	0.57 (0.37–4.98)	0.35 (0–0.88)	0.51 (0.20–1.20)	1.28 (0.67–7.99)	1.23 (0.24–6.45)	1. 66(0.49–8.23)	1.68 (0.31–8.67)
**Pertussis**	1.99 (1.37–3.16)	2.35 (1.33–3.81)	2.14 (1.27–3.06)	2.97 (2.22–5.84)	2.5 6(1.20–5.31)	2.49 (1.71–4.77)	2.50 (1.41–4.12)	2.31 (1.57–3.97)	1.98 (1.27–3.37)	4.69 (2.45–7.60)	3.67 (1.71–5.99)	2.49 (1.80–6.58)
**Tetanus**	0.54 (0.16–0.92)	0.54 (0.18–1.30)	0.54 (0.15–1.27)	0.44 (0.17–1.28)	0.48 (0.15–1.37)	0.51 (0.17–1.28)	0.39 (0.10–0.815)	0.47 (0.11–0.85)	0.62 (0.33–1.02)	0.44 (0.09–2.16)	0.65 (0.14–1.09)	0.45 (0.15–2.06)
**Measles**	1.60 (0.69–2.18)	1.4 6(0.71–2.45)	1.44 (0.86–2.08)	1.04 (0.54–2.80)	1.64 (0.67–2.23)	1.10 (0.52–2.37)	16.75 (1.69–23.25)	8.89 (1.93–16.38)	11.75 (4.16–18.77)	4.98 (0.68–14.28)	1.64 (0.67–2.23)	7.28 (4.51–12.33)
